# Organisationsethik in Einrichtungen des Gesundheitswesens

**DOI:** 10.1007/s00481-021-00639-w

**Published:** 2021-07-01

**Authors:** Christiane Burmeister, Robert Ranisch, Cordula Brand, Uta Müller

**Affiliations:** 1 Internationales Zentrum für Ethik in den Wissenschaften (IZEW), Eberhard Karls Universität Tübingen, Wilhelmstraße 19, 72074 Tübingen, Deutschland; 2 Professur für Medizinische Ethik mit Schwerpunkt auf Digitalisierung, Fakultät für Gesundheitswissenschaften Brandenburg, Universität Potsdam, Potsdam, Deutschland; 3 Internationales Zentrum für Ethik in den Wissenschaften (IZEW), Universität Tübingen, Tübingen, Deutschland

Krankenhäuser, Pflegeeinrichtungen und Hospize, ambulante Praxen und Zentren sind Orte individueller Schicksale und Begegnungen. Sie sind aber auch Institutionen mit Regelsystemen, in denen Arbeit standardisiert, Entscheidungswege und Hierarchien geordnet, Kompetenz‑, Mittel-, und Rechteverteilungen festgelegt werden. Als solche strukturieren sie die unterschiedlichen Perspektiven verschiedener Professionen, Mitarbeiterebenen und Anspruchsgruppen.

Die ethische Relevanz dieser Strukturen ist nicht allein im Horizont der ärztlichen Ethik zu erfassen. Besonders deutlich brachten dies die vergangenen anderthalb Jahre zum Vorschein: Ob es um die Zuweisung knapper Beatmungsplätze, Besucherregelungen oder die Vergabepraxis für begrenzte Impfdosen geht – eine Vielzahl ethisch relevanter Fragen infolge der Sars-CoV-2-Pandemie weist über die Mikroebene der individuellen Arzt-Patienten-Beziehung hinaus. Zugleich bieten zwar Verfügungen und Verordnungen staatlicher Akteure sowie klinisch-ethische Empfehlungen einschlägiger Fachgesellschaften eine Grundorientierung, dennoch bleibt deren Einpassung in die eigenen Abläufe und in die konkrete Versorgungssituation den Organisationen selbst anheimgestellt.

Unter dem Stichwort „Organisationsethik“ rücken Strukturen und Rahmenbedingungen von Einrichtungen mit Blick auf verantwortungsvolles Handeln ins Zentrum der Aufmerksamkeit. Wie können diese Strukturen gestaltet sein, um die Orientierung an grundlegenden Werten der medizinischen und pflegerischen Ethik zu gewährleisten? Welche Interaktions- und Kommunikationswege ermöglichen ein respektvolles Miteinander, welche behindern es? Wo eröffnen sich Räume der Reflexion auf herausfordernde und belastende Situationen des beruflichen Alltags? Wie kann individuelles Handeln im kollektiven Gesamtgefüge erfasst und bewertet werden?

Diese beispielhaften Fragen eröffnen zwei grundsätzlich unterscheidbare, wenn auch aufeinander bezogene Perspektiven, welche man grosso modo als „Organisation der Ethik“ und „Ethik der Organisation“ betiteln kann. Erstere widmet sich vorwiegend den in Deutschland seit Beginn der 1990er-Jahre entstandenen Strukturen der klinischen Ethikberatung sowie zunehmend der ethischen Reflexion in Pflege- und ambulanten Einrichtungen. Das Erkenntnisinteresse liegt bislang vornehmlich auf Struktur, Arbeitsweise und Implementierung von organisationsethischen Instrumenten wie Ethikkomitees und ethischen Fallbesprechungen sowie auf deren Wirkung bzw. Wirksamkeit. Die Entwicklung von Ziel- und Qualitätsparametern erscheint indessen noch als häufig beklagtes Desiderat.

Mit der zweiten, stärker systemischen Blickrichtung wird die Organisation in ihrer konkreten Gestalt(ung) zum Gegenstand der Reflexion. Der Fokus liegt hier auf organisationalen Rahmenbedingungen oder auf Grundsatzüberlegungen zu relevanten Konzepten und strukturprägenden Phänomenen.

Ziel des vorliegenden Themenhefts ist es, verschiedene Ansätze aus diesem breiten Spektrum organisationsethischer Reflexion vorzustellen und damit weitere Impulse für dessen Entwicklung zu geben.

Den Einstieg leistet Matthias Kettner mit einem Vorschlag, gestörte Verantwortungsrelationen in Einrichtungen des Gesundheitswesens mit neuen analytischen Begriffen zu erfassen. Die Organisationsethik, so sein Ausgangspunkt, habe es mit verschiedenartigen Belangen im Spannungsfeld zwischen ärztlichen, wirtschaftlichen und managerialen Rationalitäten zu tun. Auch wenn uns Reibungen dieser Art auf der Handlungsebene als Konflikte zwischen verschiedenen Professionsträger*innen erscheinen, so entstünden sie doch in einem organisationalen Gesamtgefüge, das die Beziehungen ihrer Mitglieder vertragsmäßig überformt und selbst von übergeordneten politischen Vorgaben beeinflusst wird. Ohne ein Verständnis solcher inter- und intrasystemischen Wechselwirkungen (bspw. zwischen Allokationsentscheidungen auf Makroebene, dem DRG-geleiteten Vergütungssystem und der vielfach beklagten Beeinträchtigung des Patientenwohls) ließen sich bestimmte Probleme weder in ihrer strukturellen Dimension erfassen, noch Lösungsansätzen zuführen. Der Autor schlägt vor, diese „Miseren“ als Folge massiv gestörter Verantwortungsverhältnisse zu begreifen und analog der medizinischen Krankheitslehre hinsichtlich ihrer Entstehungs‑, Erscheinungs- und Wirkungsweise zu analysieren. Eine um diesen Ansatz bereicherte „klinische Organisationsethik“ könne die relevanten Bedingungen, Mechanismen und Formen institutioneller Pathologien adäquater beschreiben und ihnen proaktiv begegnen („Miseren des Krankenhauses, institutionelle Pathologien und klinische Organisationsethik“).

Um eine ganz konkrete „Misere“ dreht sich der Beitrag von Karl-Heinz Wehkamp, der die Ergebnisse einer 2017 veröffentlichten Studie zu jüngeren Veränderungen im deutschen Gesundheitswesen organisationsethisch rekapituliert (Wehkamp und Naegler 2017). Wie aus qualitativ analysierten Interviews mit Vertreter*innen der Ärzteschaft und der Geschäftsführung klinischer Organisationen hervorgeht, gehen die Perspektiven dieser Personengruppen erheblich auseinander, wenn es um Ausmaß und Einfluss betriebswirtschaftlicher Vorgaben auf die Behandlung von Patient*innen geht. So führten Kostendruck und Effizienzvorgaben nach Auffassung vieler Ärzt*innen zunehmend zu ethisch fragwürdigen Entscheidungen sowie falschen Behandlungen, Unter- oder Überversorgung. Dagegen bestätigten Geschäftsführer*innen zwar die finanziell angespannten Rahmenbedingungen, nahmen jedoch keine negativen Auswirkungen wahr. Dass diese Ergebnisse wiederum selbst polarisieren, legt eine abschließende Schilderung der Reaktionen auf die Studie nahe. So stünden den spürbaren Widerständen auf Seiten der Wissenschaftspolitik ein beträchtliches Medieninteresse und die überwiegende Zustimmung auf Seiten der Ärzteschaft entgegen. Letztere sehen die Möglichkeit zur Verbesserung ihres Arbeitsalltags vor allem in einer offeneren Kommunikation und Auseinandersetzung über die aktuellen Entwicklungen in den Krankenhäusern („Medizinethik und Ökonomie im Krankenhaus – die Kluft zwischen Anspruch und Wirklichkeit. Ergebnisse einer qualitativen Studie“).

Was hier in Ansätzen gefordert wird, untersucht Georg Marckmann in seinem Beitrag systematisch: die Möglichkeit, den Bedingungen der „Mangelverwaltung“ (Marckmann und Maschmann 2017) mit organisationsethischen Mitteln entgegenzusteuern. Zunächst erhärtet sein Überblick über die empirischen Befunde den Verdacht der potenziell schädlichen Auswirkungen ökonomischer Vorgaben im Krankenhaus. Zugleich nimmt der Autor jedoch Spielräume für eine Verbesserung der Arbeitsorganisation und Zusammenarbeit sowohl auf struktureller als auch auf kultureller Ebene wahr. Sie böten organisationsethische Handlungsperspektiven, aus denen das Grundgerüst eines Wertemanagements generiert werden könne. Als ersten Schritt schlägt der Autor vor, die für ein Krankenhaus relevanten, durchaus auf verschiedene Akteure der Organisation bezogenen ethischen Werte offen mit allen Mitarbeitenden herauszuarbeiten. Anschließend sind im Besonderen die Führungskräfte der Organisation angehalten, die tatsächliche Orientierung an diesem Wertgerüst zu etablieren und u. U. Arbeitsabläufe anzupassen. Dabei setzt der Autor auch auf die vielfach bereits vorhandenen Klinischen Ethikkomitees und entwickelt ein Grundmodell einer Fallanalyse mit organisationsethischer Ausrichtung und Interventionskompetenz („Ökonomisierung im Gesundheitswesen als organisationsethische Herausforderung“).

Cordula Brand wiederum erhellt mit ihrer kritischen Betrachtung des aus der Unternehmensethik stammenden Konzepts „Diversitätsmanagement“, inwiefern organisationsethische Instrumente auch selbst Ergebnisse ökonomischer Engführung sein können. Wie die Autorin beschreibt, wird Diversitätsmanagement gemeinhin als ein Schlüssel gesehen, um in Anbetracht einer globalisierten Welt auch als soziale Organisation wettbewerbsfähig zu bleiben, wirtschaftlich zu arbeiten und der steigenden Vielfalt am Arbeitsplatz sowie am Patientenbett gerecht zu werden. Die Autorin diskutiert einige Fallstricke einer solchen Übertragung und stellt dabei heraus, dass diese Gefahr läuft, Effizienz auf ein rein wirtschaftliches Verständnis zu reduzieren. Wolle es seine Aufgabe ernst nehmen, zur sozialen Gerechtigkeit beizutragen, so bedarf das Diversitätsmanagement eines Gesamtkonzepts hinsichtlich der zu berücksichtigenden Diversitätsdimensionen sowie einer substanziellen Beteiligung aller Betroffenen und schließlich der Anwendung des Konzepts in allen organisationalen Strukturen. Wie Brand resümiert, sollten bei der Abwägung und Evaluation von Maßnahmen Kosten‑/Nutzen-Überlegungen stets in den Dienst der Gerechtigkeit gestellt werden, nicht umgekehrt („Diversitätsmanagement in Organisationen des Gesundheitswesens – Effizienz kontra Gerechtigkeit?“).

Neben den ökonomischen Rahmenbedingungen prägt zunehmend auch die Digitalisierung Strukturen und Prozesse in Organisationen des Gesundheitswesens. Ihren moralisch relevanten Auswirkungen auf Entscheidungsprozeduren sowie den damit verbundenen Fragen widmet sich Arne Manzeschke. Gerade die Blickrichtung auf das System „Organisation“ erlaube jenen angemessenen Zugang zu kollektiven Entscheidungen und organisationaler Wechselwirkungen, der erklären kann, inwiefern digitaler Wandel die Wissensbestände, Heuristiken oder Praktiken verändert, neue Handlungsfelder und Kommunikationsräume kreiert und Veranwortungsrelationen verwässert. Mit sowohl organisationsethischen als auch technikphilosophischen Beschreibungen betrachtet der Autor verschiedene Aspekte und Ebenen ein und desselben Kernproblems: die Desintegration des Menschen als moralischen Akteur. Damit fügt Manzeschke den bisherigen Zielbestimmungen der Organisationsethik eine entscheidende Aufgabe hinzu: die systemisch informierte Analyse digitalisierter Prozesse unter dem Gesichtspunkt der gerade in Einrichtungen des Gesundheitswesens unverzichtbaren menschlichen Autorschaft („Digitalisierung und Organisationsethik. Ethische und technikphilosophische Skizzen“).

Das Problem, dass ebenjene Autorschaft nicht nur Ursprung wohlüberlegter Entscheidungen, sondern auch Quelle menschlichen Irrtums ist, liegt dem Beitrag von Kurt Schmidt zugrunde. Ausgangspunkt des Autors ist der evidente Umstand, dass die Konfrontation mit schweren Krankheiten, Sterben und Tod in Einrichtungen konzentrierter medizinischer Versorgung wie Kliniken zum Berufsalltag der Mitarbeitenden gehört und diese an die Grenzen ihrer Belastbarkeit bringen kann. In diesem Kontext stellt, so Schmidt, der Umgang mit (mutmaßlichen) Behandlungsfehlern eine besondere Herausforderung dar. Wie Organisationsethik hier hilfreich eingesetzt werden kann, liegt im Fokus des Beitrags. Nach einer Analyse verschiedener Konsequenzen und zu bedenkender Problemstellungen in Fällen mutmaßlicher Fehler werden die Möglichkeiten von Kliniken erörtert, Mitarbeitende auf verschiedenen Ebenen zu unterstützen. Für den Autor ist dabei nicht vorrangig die, gleichwohl sehr wichtige, Prävention von Behandlungsfehlern von Interesse, sondern die Bemühung, Mitarbeitende als potenzielle „second victims“ nicht im Stich zu lassen und einen klärenden, wertschätzenden und strukturierten Umgang mit ihnen zu etablieren („Der Umgang mit belastenden Ereignissen als organisationsethische Herausforderung am Beispiel ‚Behandlungsfehler‘“).

Wie Patrick Schuchter, Thomas Krobath, Andreas Heller und Thomas Schmidt verdeutlichen, lassen sich Lernprozesse dieser Art weder in der Logik von Zweck-Mittel-Relationen noch in der einfachen Metapher eines „lernenden Organismus“ adäquat erfassen. Die Autoren verstehen Organisationen des Gesundheitswesens als komplexe soziale Systeme mit jeweils eigenen (formalen wie informalen) „Spielregeln“ der Mitgliedschaft, Programmgestaltung und Kommunikation, welche nicht nur die spezifischen Sichtweisen und Interessen der Beteiligten prägen, sondern auch entscheidend beeinflussen, welche ethischen Fragen überhaupt gesehen werden. In dieser für jede Organisationsform einzigartigen Perspektivenvielfalt müssten die besonderen Kultur- und Strukturmerkmale sowie der Sinn der Organisation selbst beständig reflektiert werden. Mit einem kurzen Rekurs auf die Geschichte der Organisationsethik begründen die Autoren deren zentrale Orientierung an (1) der gemeinsamen Verantwortungsübernahme von klinischer Ethik, Professionsethiken und Ökonomie sowie (2) der Organisation von Verständigungsprozessen und (3) transdisziplinär gestalteten Lernprozessen. Auf dieser Basis werden schließlich drei Kompetenzbereiche skizziert, die für eine prozessorientierte Organisationsethik im Gesundheitswesen entscheidend sind: die Schaffung und Sicherung von Reflexionsräumen im organisationalen Alltag, die Offenheit für verschiedenste Diskussionsweisen an Fragen des „Guten“ bzw. „guten Lebens“ sowie die Verknüpfung der verschiedenen Reflexionsräume zu einer für die Einrichtung passenden Gesamtarchitektur („Organisationsethik. Impulse für die Weiterentwicklung der Ethik im Gesundheitssystem“).

Wie solche Unterfangen in der Praxis aussehen können und welche konzeptionellen und implementierungstechnischen Fragen sich mit ihnen verbinden – kurz: wie die Organisation von Ethik (besser) gestaltet werden kann –, wollen die beiden abschließenden Beiträge mit Schwerpunkt auf jeweils unterschiedliche Organisationsformen beispielhaft beleuchten.

Im ersten Projektbericht stellen Robert Ranisch, Annette Riedel, Friedemann Bresch, Hiltrud Mayer, Klaus-Dieter Pape, Gerda Weise und Petra Renz ein Pilotprojekt vor, das auf allen Stationen des Universitätsklinikums Tübingen den Einsatz geschulter Pflegekräfte als Ansprechpartner*innen für ethische Fragen vorsieht. Das multidisziplinäre Autorenteam nimmt kritische Stimmen gegenüber etablierten Formen klinischer Ethikberatung als wenig wirkmächtige „Expertengremien“ zum Ausgangspunkt, um die Limitationen des eigenen, bisher implementierten Modells, besonders hinsichtlich der Inanspruchnahme durch Pflegende, offenzulegen. Vor diesem Hintergrund werden Zielsetzung und Konzept der „Ethikbeauftragten auf Station“ vorgestellt, welche das bestehende Angebot um dezentrale Strukturen und die aktive, mehrfache Einbindung der pflegerischen Berufsgruppe erweitert („Das Tübinger Modell der ‚Ethikbeauftragten der Station‘: Ein Pilotprojekt zum Aufbau dezentraler Strukturen der Ethikberatung an einem Universitätsklinikum“).

An- und insgesamt abschließend wird mit dem Projektbericht von Christiane Burmeister, Ariane Iller, Robert Ranisch, Cordula Brand, Tobias Staib und Uta Müller ein Blick auf das Ethikmanagement in dezentral organisierten Einrichtungen des Sozial- und Gesundheitswesens geworfen. Ihr Beitrag stellt das interaktive, auf dem Nijmegener Modell basierende Ethikkonzept der BruderhausDiakonie Reutlingen vor, deren Einrichtungen nicht nur räumlich weit verstreut sind, sondern von der Sozialpsychiatrie bis zur Behindertenhilfe verschiedene professionelle Handlungsfelder unter sich vereinen. Da unter diesen Bedingungen die Zusammenarbeit zwischen zentralen und dezentralen Ethikstrukturen sowie top-down- und bottom-up-förmigen Reflexionsweisen nicht vorausgesetzt werden kann, haben die Autor*innen mit der Position der/des Ethikbeauftragten ein Verbindungselement entwickelt, das die Umsetzung des Nijmegener Modells auch in Organisationen „jenseits der Klinik“ ermöglichen soll („Jenseits der Klinik: Konzeptionelle Überlegungen zum Ethiktransfer in dezentralen Einrichtungen des Gesundheitswesens am Beispiel der BruderhausDiakonie Reutlingen“).
